# A practical approach to low protein diets in Sweden– 45 years of clinical use

**DOI:** 10.1186/s12882-016-0295-6

**Published:** 2016-07-19

**Authors:** Sintra Eyre, Gerd Faxén-Irving, Per-Ola Attman, Marie Evans, Karin Windahl, Sigrid Wegener, Charlotte Andersén, Karin Nykvist-Raanaes, Sara Einemo, Juan-Jesus Carrero

**Affiliations:** Department of Clinical Nutrition, Sahlgrenska University Hospital, 413 45 Gothenburg, Sweden; Division of Clinical Geriatrics, Department of Neurobiology, Care Sciences and Society, Karolinska Institutet, Karolinska, Sweden; Department of Nephrology, Sahlgrenska University Hospital, Gothenburg, Sweden; Division of Renal Medicine (CLINTEC), Karolinska Institutet, Karolinska, Sweden; Department of Clinical Nutrition, Academic University Hospital, Uppsala, Sweden; Department of Nephrology, Skåne University Hospital, Malmö, Sweden; Department of Clinical Nutrition and Dietetics, Karolinska University Hospital, Karolinska, Sweden

**Keywords:** Chronic Kidney failure, Nutrition therapy, Diet, Protein restriction, Dietary proteins

## Abstract

This review provides an overview of the development, implementation and practise of low protein diets (LPD) in Sweden. While the current practice is discussed in general terms emphasizing the interplay between nephrologists and dieticians, the ”self-selected” LPD model is explained as a practical approach to facilitated patient’s adherence to the nutritional therapy. This model is currently implemented in most clinics of the country and gives considerable flexibility regarding variation in meal planning, food selection, amounts consumed, cooking methods as well as adaptations to day-to-day changes. Current LPD use in Sweden is presented through analysis of the Swedish Renal Registry. Finally two patient cases are illustrated, with examples on their diets, attempts to reduce the protein content to the desired thresholds and their clinical course.

## Introduction

The use of low protein diets (LPD) as a treatment for patients with renal failure based on the work of Giovannetti and Maggiore [1] started in Sweden in the beginning of 1970’s. LPD was adapted and modified to suit the food-habits in Sweden. Given the seminal work of some foremost researchers on the development of LPDs and its implementation in the country, their ideas and imprints still prevail, justifying a brief historical contextualization. In this review we provide a historical overview on LPD use in Sweden, followed by an analysis of the current situation as reported in the Swedish Renal Registry. In order to allow comparison with other countries, the clinical practicalities regarding patient education, diet design and patient follow-up are discussed through questions pre-specified by the editors of this issue. The terms nutrition therapy, diet and LPD have been used in this review interchangeably. Finally, we provide two patient cases with examples on their diets, attempts to reduce the protein content to the desired threshold and their clinical course.

## Historical development

A Swedish group led by the late Professor Jonas Bergström at Karolinska Institutet in Stockholm [2], decided to explore the possibility of improving the quality of the low protein diet by supplementing it with essential L-amino acids. They showed that nitrogen balance was neutral or even positive with the use of amino acid (AA) solutions containing the 8 essential amino acids (EAA) and histidine compared to solutions containing only the 8 EAA [3, 4]. To avoid metabolic acidosis the basic AA was provided as acetate salts, since acetate is metabolized to bicarbonate. A solution containing the essential L-amino acids in proportions as originally proposed by Rose [5] was developed in co-operation with KabiVitrum AB Sweden and commercially distributed as Aminess®. It was initially given intravenously and was successful for short-term treatment of uremic patients. Coated EAA-tablets that did not dissolve in the mouth were developed thereafter and a diet regime with 18 g protein supplemented with 20-30 tablets/day was introduced in the clinics. Later it was found that such high doses of EAA were not really necessary and that 12-20 tablets/day was sufficient for most patients [2]. The LPD supplemented with this formulation was initiated as the standard treatment for patients with near-end-stage renal failure in Sweden. The diet was also successfully applied in other countries, especially Germany.

Professor Jonas Bergström and Professor Anders Alvestrand *et al.* continued their work on AA-metabolism in renal failure and reported findings of low intracellular concentrations of threonine, valine, lysine and tyrosine and increased concentrations of several non-EAA in untreated uremic patients [2, 6, 7]. Some of these abnormalities persisted in spite of long-term treatment with a LPD (16-20 g protein/day) supplemented with the EAA formulation. A new EAA formula with changed contents and proportions was designed to correct these abnormalities. The new preparation (Aminess-N®) contained proportionally more valine (+70 %] and less leucine and isoleucine than prescribed by the Rose formula [5], and in addition histidine and tyrosine. Studies with this new preparation showed that abnormalities of EAA in plasma and muscle characteristic of uremia can be corrected by nutritional means, and that uremic patients require AA in other proportions than healthy subjects [8]. This preparation is still in use.

The late Professor Härje Bucht from Karolinska Hospital moved to Gothenburg in 1970 and established the treatment with LPD at the Sahlgrenska university Hospital. During the following decade much research was devoted to explore the metabolic effects of the supplemented LPD. The work was mainly carried out by Professor Per-Ola Attman and included studies of nitrogen balance and body composition in patients with uremia [9]. During this period keto-analogues to the EAA were developed in Germany to further reduce the nitrogen intake. Nitrogen balance studies indicated that keto-acids could substitute for conventional EAA and were well tolerated by the patients [10]. This practice was not strongly established in Swedish clinical practice mainly because these supplements were not subsidized. The composition of the LPD in itself, with large amounts of saturated fat and simple carbohydrates, prompted an extensive study of the effects on the deranged lipid and carbohydrate metabolism that continued into the 1990’s. Despite the composition of the LPD, there was no further deterioration of the renal dyslipidemia [11].

Until the beginning of the 1980’s the patients were introduced to the LPD as in-hospital patients during a period of 1-2 weeks and assigned protein levels of 0,3 g (very low protein diet, VLPD) or 0,6 g (LPD) of protein per kg body weight/day, supplemented with EAA, iron and multivitamin tablets daily [2]. This practice gradually changed over time. Instead of starting LPD in-hospital, the patients were referred to a renal dietician by a nephrologist. By the end of the 80’s the use of LPD declined partly because access to dialysis treatment became increasingly available. There was also an opinion among some nephrologists that LPD might expose patients to the risk of developing malnutrition and that dialysis should be preferred. The concept of early dialysis or healthy start was launched and heavily backed up by industry. Other concerns were also raised, such as feasibility and cost-effectiveness. Management of patients with advanced renal failure only with diet, the so called conservative treatment, was viewed as too time-consuming by requiring close management with frequent visits in addition to patient education and physician’s and dietician’s efforts to maintain their motivation. An on-going debate persisted on whether LPD could affect the progression of renal function and/or delay the time before starting active treatment. This hypothesis was based on animal experiments and resulted in large interventional multicentre studies among which the MDRD study [12] is best known. Although the results of the study were interpreted differently depending on attitude and knowledge among nephrologists, it spurred the interest of nutritional management in renal disease.

Despite these issues, LPD continued to be in use, as its advocators saw the diet as a clinically safe treatment with many benefits if implemented correctly according to guidelines on energy intake and protein quality. However, the use of VLPD has declined in the last years, and has not been in frequent use in Sweden since the 1990’s. Instead, an individualized LPD is often implemented at levels of 0.6 – 0.7 g/kg/day according to current national guidelines [13, 14]. The diet is supplemented with EAA if the total protein intake is less than ≤0.6 g/kg or if the requirement of at least 60 % high quality protein is not met. LPD is generally introduced to the patient at an out-patient encounter by a specialized dietician upon referral from a nephrologist. The treatment is put into practise in the context of the multidisciplinary teamwork of dieticians, nephrologists and nurses aiming to alleviate uremic symptoms, maintaining or improving nutritional status, preventing malnutrition and delaying the need of dialysis.

## The key – dedicated work by dieticians

In Sweden, dietician is a protected professional title with three years of higher education and licensed to practice by the National Board of Health and Welfare. Dieticians working at University hospitals become specialists in renal nutrition if they hold full-time positions in renal departments. Dieticians working in regional and local hospitals usually work generically. In addition, dieticians can attend special courses and meetings on updates in renal nutrition. The Swedish dieticians working in renal units have taken an active role in the practical implementation of LPD since the 1970’s. The first cookbook with recipes and cooking recommendations for patients and their relatives/caregivers in order to comply with a LPD was published in 1973 [15]. The authors were the dieticians Marianne Ahlberg and Marianne Wessman, one nephrologist and one patient, all at St Eriks Hospital in Stockholm. This cookbook made it easier for patients to comply with the treatment, for the staff to instruct the patients and for cooking staff in the hospital kitchens to prepare LPD meals all over Sweden. The goal for the daily protein intake was 15-20 g of protein and the recommendation for energy intake was high. The diet was supplemented with EAA as detailed earlier. Unsalted butter and cream was recommended in cooking to ensure that the energy requirements were met. No sodium was added. Vegetables were included in small amounts, which probably reflect the eating habits in the Swedish society at that time. Low protein products such as pasta and bread were, and still are available for the patients and subsidized by the government.

A second cookbook was published in 1977 [16]. This contained menu suggestions and inspiration to achieve different levels of protein intake of 20, 40, and 60 g/day. It still recommended no additional sodium, small portions of vegetables and increased intake of fat from unsalted butter and cream sources. This book was widely spread throughout the country and most clinics used the recipes for planning and cooking as well as to train the patients. During a few years, at Huddinge hospital in Stockholm, patients were given the option to purchase 1 to 3-weeks of ready-made frozen low-protein dishes to bring home and ease compliance.

A third cookbook was published in 2002 by two dieticians at Sahlgrenska University hospital in Gothenburg [17]. This book is still in use and available for purchase, and two easy recipes are here provided as an example (Table 1). The cookbook was published as an addition to the development/creation of a “self-selected” LPD model for the practical implementation of LPD developed by the renal dietician Gunilla Uddebom in the late 1980’s [18]. This LPD model was initiated due to an observed desire from the patients to take a more active role in the planning and implementation of the diet. The model was introduced on full scale at the Sahlgrenska University hospital in 1988 accompanied with a patient-oriented manual to be handed out to patients prescribed with LPD. The main purpose was to enable the adaptation of the diet to the patients’ own habits at home, in a family setting, at work, on travels or in leisure time. The ”self-selected” LPD model gives considerable flexibility regarding variation in meal planning, food selection, amounts consumed, cooking methods as well as possibilities for day-to-day changes of the whole diet plan with options for most foods. The goal is to educate the patient on how to plan their diets in order to achieve an intake of 0,6 g protein /kg/day and an energy content of 35-40 kcal/kg/day. The manual includes an introduction to the diet followed by information on food items divided into ten different groups (Table 2). The food groups are identified according to Swedish food traditions and use. Cheese is found in the group of spreads and toppings as this food item is mainly eaten as a topping on bread in Sweden. Hence, there needs to be a guide on how cheese can be exchanged with other toppings and spreads such as thin slices of ham or sausage. Butter on the other hand is grouped together with fats and oils in a food group which is as good as free from protein and therefore free to eat. Included in the group “dairy products” is e.g. milk (which is a common to drink together with cooked meals), yoghurt, cream and ice cream but also oil or oat- based “dairy-like” substitutes for milk and sorbet as a substitute for ice cream.

**Table 1:** Examples of low-protein diet cooking recipes for patients with CKD

Pasta gratin with spinach, champignon and bacon (Serves 2)
*Ingredients* 3 dl uncooked low protein pasta Water + a pinch of salt *or* bullion 125 g frozen baby spinach 1 pinch of salt ¼ teaspoon ground nutmeg 100 g bacon (approx. 10 slices) 100 g sliced champignons Margarine or butter 2 x ½ dl half-and-half or cream 2 x 10 g shredded cheese containing at least 39 % fat
*Directions* Turn the oven to 225 °C. Cook the pasta according to direction on the package in water or bullion. Drain thoroughly. Defrost the spinach, press out excess fluid and add salt and nutmeg. Dice the bacon, fry and remove from the pan. Fry the sliced champignons in the fat from the bacon, add margarine or butter if needed. Grease two 1-portion sized ovenproof gratin dishes. Coat the bottoms with the boiled pasta. Divide spinach, champignons and bacon evenly. Pour ½ dl cream over each portion and drizzle the grated cheese on top. Cook in oven for 20 minutes. Serve with a mixed salad, bread and butter.
*Protein content:* 1 portion contains 13 grams of protein
Warm apple compote with cinnamon and ice cream (Serves 2)
*Ingredients* 200 g peeled and seeded apple (approx. 2 apples) 1-2 tablespoons of butter 1 tablespoon honey 1 teaspoon cinnamon 2 x 50 g ice cream containing a least 12 % fat
*Directions* Slice the apples. Melt the butter in a saucepan and stir in the honey. Add apple slices and boil them slowly until soft on low heat. Divide evenly in two portions. Serve with ice cream and dust over cinnamon.
*Protein content: 1 portion contains 2 grams of protein*

**Table 2:** Food groups used in the “self-selected” low protein diet model

1. Meat and poultry	
2. Fish, shellfish and egg	
3. Potatoes, rice and pasta	
4. Vegetables	
5. Bread and cereals	
6. Ham, cheese, spreadings, cold cuts etc.	
7. Dairy and “dairy-like” products	
8. Fruits and berries	
9. Beverages	
10. Margarines, butter, oils, marmalades etc.	

**Fig. 1:**
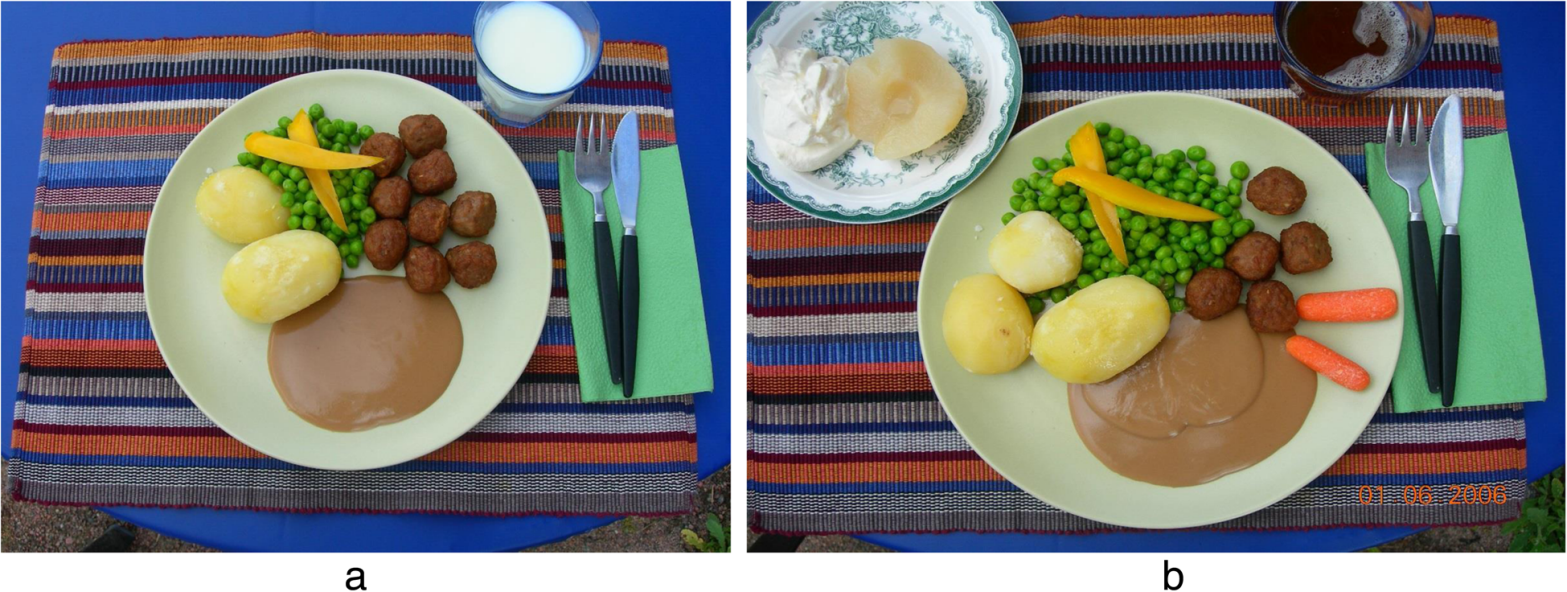
A normal portion size and its adaptation to a LPD portion size. The left plate (**a**) shows a normal portion size with traditional Swedish food. In the right plate (**b**), the portion has been adjusted to a LPD according to the “self-selected” model; i.e. milk has been exchanged to a berry drink, less meat, more potato, sauce and vegetables as well as a dessert with canned fruit and whipped cream

Each group contains a fixed amount of protein per serving with the exception of two groups, which are almost free from protein but provide considerable amounts of energy, principally from unsaturated fats and to some extent from saccharides. The amount of protein per serving is equal within each food group irrespective of the food selection (see example in Table 3). The patient decides upon the number of servings from the food groups within the limits of the described daily protein intake. They are advised to distribute the protein intake over the day and to choose food items from all 10 food-groups in order to get a well-balanced diet. The manual is available for purchase through the website of the Swedish Association of Clinical Dieticians’ Reference group in Renal Nutrition. The self-selected model is schematically illustrated by photos (Fig. 1) of a normal portion size of a traditional Swedish meal and its adaptation to a LPD portion size of the same meal using the manual with suggested amounts of foods from the different food-groups.

**Table 3:** Examples of food choices in the “self-selected” low protein diet model

Examples from food group 1 and 2 (containing 10 g protein/serving]	Serving, g meat, fish or egg	Examples from food group 3 and 5 (containing 2 g protein/serving	Serving, g potatoes, rice, pasta and bread and cereal
Meat, lamb, pork, veal	50	Potatoes	100
Chicken	50	Mashed potatoes	100 (1 dL)
Minced meat	50	French fries	70 (1,5 dL)
Sausage	90	Rice, uncooked	30 (3 tbs)
		Rice, cooked	90 (1 dL)
Fish	50	Pasta, uncooked	15 (1,5 tbs)
Herring, pickled	80	Pasta, cooked	45 (3/4 dL)
Sardines in oil	40	Low protein rice	free
Tuna in oil, canned	30	Low protein pasta	free
Mussels, canned, drained	60	Bread	25 g (1 thin slice)
		Crisp bread	25 (2 thin slices)
*6 g protein/serving*		Oatmeal	3 tbs
Egg	50		

*Abbreviations*: *dL* deciliter, *tbs* table spoon

The model has been in use for nearly 30 years in more than five hundred patients at the Sahlgrenska University hospital. It has been evaluated in a retrospective case-control study including 122 patients [19]. The evaluation showed that treatment with LPD according to the self-selected model was not inferior (with respect to the risk of morbidity during the first year in dialysis and mortality), and in several instances superior to conventional pre-dialysis care without LPD (better nutritional status achieved and less morbidity at the time of starting dialysis). A systematic review of body composition in LPD was undertaken [20] to strengthen the clinics view of LPD as a clinically safe and effective treatment for patients with CKD.

Since 2004 a handbook has been available in Sweden with the title “Living with renal failure” developed by the patient Per-Åke Zillén and supported/reviewed by various healthcare professionals. The aim of this handbook is to assist CKD patients in their everyday life. One full chapter is devoted to the protein content of different foods in portions, pieces, cups and glasses in order to facilitate a daily overview of the protein intake. New editions have been released in 2007, 2011 and in 2014 [21].

## The use of LPD in Sweden today

Since 2008, incident patients with CKD 4-5 followed by a nephrologist are registered into the Swedish Renal Registry Chronic Kidney Disease (SRR-CKD). Currently, non-dialysis data collection of the SRR includes more than 95 % of all out-patient nephrology care and representativeness is estimated to range from 75-90 % during 2015 (www.snronline.se). During data collection, it is mandatory to report on whether the patient is treated with a LPD. The definition of LPD stated as per protocol of data collection is a prescription of 0.6 g /kg body weight/ day. Other rates of protein restriction may exist, but are not currently considered in the registry.

**Fig. 2:**
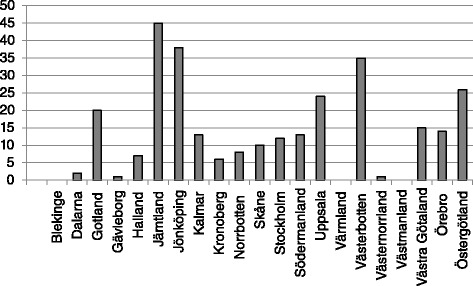
Proportion of patients (%) with low-protein diet in stage 4-5 CKD per county

We accessed the SRR to evaluate the extent of LPD use among CKD patients. This was performed using all patients registered with an eGFR <45 ml/min/1.73 m^2^ during the time span ranging from June 2013 to June 2015. In total, 10 % of these patients were treated with a LPD ≤0.6 g/kg. About half of the Swedish clinics (51 %) reported to use LPD as defined by the Registry whereas the rest of the centres used it in very few patients or not at all. According to the registry, 18 % of the clinics use it in more than 20 % of the patients. The choice of LPD treatment is more available in larger hospitals, including regional hospitals (12.7 % of LPD use) and university hospital (10.2 %), as opposed to local hospital (7.0 %). The use of LPD also differed within the Swedish counties and regions, being more common in patients with CKD stage 4-5 among the counties of Västerbotten (38 %), Jämtland (45 %) and Jönköping (35 %) (Fig. 2). LPD was more commonly used in the north and southeast parts of Sweden, but the use seemed more to reflect local traditions as it could vary also within the same county or region.

As a next step we attempted to evaluate patient characteristics in centres using LPD (defined for this purpose as centres with >5 % of patients following LPD in CKD stage 4-5). This involved 25 centres and 5997 patients. We observed that LPD was more commonly followed in individuals with CKD stage 5 (39 % use), followed by CKD stage 4 (14 %) and CKD stage 3 (8 %). There were no major differences in the use of LPD overall based on sex (men 18.5 %, women 17.1 %), and LPD use was relatively similar through different age strata. If we focused on the patient group where a LPD is recommended (eGFR <20 ml/min/1.73 m^2^, *n* = 2391) we observed that patients diagnosed with adult polycystic kidney disease and glomerulonephritis (41 and 38 % respectively) were prescribed LPD more often than most other patient categories, while those with non-determined renal disease were prescribed LPD less often (22 %). Also patients with a body mass index (BMI) ≤20 kg/m^2^ were prescribed LPD less often compared with patients of normal or high BMI. Presence of comorbid diseases such as diabetes, ischemic heart disease or cerebrovascular disease was associated with a lower use of LPD in general. Adjusting for age, sex, primary renal disease, body mass index and co-morbid diseases, the only factor that was independently associated with a higher use of LPD was normal BMI (20-25 kg/m^2^) Risk Ratio (RR 1.2; 95 % confidence interval [CI] 1.0-1.4) while both unknown renal disease and ischemic heart disease was significantly associated with a lower use for LPD (RR 0.65; 95 % CI 0.5-0.9 and RR 0.74; 95 % CI 0.6-0.9 respectively).

## Practical implementation process

The Swedish Association of Clinical Dieticians’ Reference group in Renal Nutrition is comprised of seven board members, each working in different regions of Sweden. The group has initiated and developed patient material for the nutritional care of adult pre-dialysis patients and has co-operated with the Swedish Kidney Association in developing national guidelines for good nutritional care in renal disease. Below follows a consensus view of current practical implementation processes of the LPD in Swedish practice involving:

1. What kind(s) of diet(s) do you prescribe in your Center-Unit?
  The most common prescription is 0.6 g protein/Kg/d but also 0.7-0.8 g/ Kg /d. The use of VLPD is rare.
2. Do you allow unrestricted meals? If yes, how many per week?
  Generally, there is no specific information/recommendation on unrestricted meals as a planned part of the LPD treatment. Eating more freely during special occasions is allowed as long as he or she returns to the recommended level of protein restriction thereafter.
3. Who is the main prescribing physician of the diet?
  The LPD is initiated by a nephrologist and generally introduced to the patient by a renal dietician at the out-patient department in close collaboration with the nephrologist and nurses. A key factor to success with LPD is to count with nephrologists that have the competence and interest on LPDs.
4. Which patients do you include in a dietary program?
  Generally, patients with GFR ≤20 ml/min, CKD 4-5 and uremic symptoms are included in the dietary program.
5. Which patients do you exclude from the study diet and from all diets?
  All patients are individually assessed regarding their ability to comply with a LPD. Old patients with comorbidities, patients with dementia, malnutrition, patients with psychological or social problems that make it difficult to comply with LPD are excluded.
6. Which clinical and laboratory tests do you use before starting the diet and which tests do you routinely prescribe in on-diet patients?
  Routine clinical and laboratory tests that determine the LPD initiations and its monitoring are generally urea, creatinine, potassium, phosphate, albumin, glucose, CRP and bicarbonate in plasma. 24-hour urinary urea nitrogen appearance (UNA). GFR according to cystatine-C and eGFR or creatinine clearance. Iohexol clearance is optimal but not routinely used. Pt-U alb/protein. Weight, BMI, body composition measures if possible.
7. How is the out-patient clinic for CKD patients organized?
  The outpatient clinic and follow up is based on an individual-patient basis, but generally scheduling a minimum of 4 visits/year with a dietician over and above the visits to the physician. Upon progression to CKD stage 5 the visits become more frequent. In between visits, regular follow-ups are performed through telephone interviews, which on average span for 15-45 minutes. In many out-patient centers there are renal coordinators that try to schedule coordinated patient-centered visits gathering the dietician/nurse/doctor at the same time. Many clinics have, in addition, educational sessions for both patients and relatives (3-4 sessions). In general they include: a session about basic kidney function and CKD held by a physician, one session about basic nutrition, LPD in theory and practice by a dietician, one session about what support the patient and relatives can get (economy and therapeutic talks) by a social worker. Finally a patient based session - two patients give their experience of treatment with peritoneal dialysis and hemodialysis. The sessions are given in the afternoon and last about 2 hours.

### First visit to the dietician

Information is given on rationale of engaging into a LPD, the need of nutritional assessment and monitoring as well as the importance of 24-hour dietary recalls for estimation of energy- and protein intake. The patient is provided with written material and advice on selection and avoidance of specific food items according to individual needs. The patient thereafter gets a personal menu based on the prescribed amount of protein/kg/day.

Patients on LPD (≤0.6 g protein/Kg/d) are generally supplemented with commercially available EAA formulas. They are also supplemented with water-soluble vitamins (B, C and B12 if needed). Vitamin D is prescribed by the physician.

If oral intake or energy intake is assessed as unsatisfactory, low protein oral supplements and/ or energy formulas (fat, carbohydrate) are prescribed by the dietician. Low protein pasta, cereal, crackers and bread are also possible to prescribe and partially tax-funded (rules differ within the country).

### Follow-up in clinical practice

The dietician evaluates adherence to the nutritional therapy in collaboration with nephrologist through monitoring of weight change, laboratory values, 24-hour urinary urea nitrogen appearance (UNA) and 24-hour dietary recalls for estimation of energy- and protein intake as well. Food diaries are often completed when needed.

## Patient case reports

Two patient case reports are presented below.

### First patient case

One of the longest followers of LPD reported from Sahlgrenska University Hospital is a man born in 1933 with the diagnosis renovascular hypertension. In 1998 he asked his nephrologist if there was anything he could do to possibly slow down the decline of his renal function. As he was a particularly compliant patient, the nephrologist proposed LPD, even though he had no uremic symptoms and his eGFR was considerably higher (30 ml/min) than the general level when LPD is usually started in that clinic (<20 ml/min). This proposal was inspired by the meta-analysis of Pedrini et al. suggesting beneficial effects of LPD even at early stages of CKD [22].

The patient started LPD following a menu of 45 g protein/day equaling 0,6 g protein/kg/day with great support from his wife, who has been the chief cook in the family during the whole time. The diet plan was based on his food-habits with adjustments done to reduce the protein intake accordingly (Table 4). He has had regular follow-up (initially every 3 months, during his stable period every 6 months thereafter and since GFR has dropped below 15 ml/min again every 3 months), with clinical evaluations detailed in Table 5. He has regularly provided his food diaries reflecting the choice of low protein foods and water soluble vitamins and periodically using vitamin B_12_ and magnesium. He has also been physically active with 1-2 hours of brisk walking every day and work in the garden.

**Table 4:** Suggested dietary plan to achieve a protein intake of 45 g per day following the “self-selected” LPD model on the basis of the patient’s reported food records

Time		Food group	Serving	Protein, g
04.30	Crisp bread, 1 slice	5	½	1
	Rice cake	5	1/3	0,5
	Margarine, use generously	10	n.r	0
	Sausage, 2 thin slices (in total 20 g)	6	1	2
	Cucumber, tomato, parsley – a few slices	4	<1	<1
08.00	Yoghurt	7	1 ½	4 ½
	Cereals	5	1	2
	1 slice of bread	5	1	2
	1 slice of crisp bread	5	½	1
	Margarine, use generously	10	n.r	0
	Sandwich spread	6	1	2
	Cucumber, tomato, parsley – a few slices	4	<1	<1
	Apple	8	1	1
10.00	Coffee	9	n.r	0
	Cookie, cracker or bun	5	1	2
12.30	Potato, rice or pasta	3	2	4
	Meat, chicken or fish	1 or 2	1	10
	Margarine or oil for cooking	10	n.r	0
	Cream, crème fraiche or half-n-half (for sauce)	7	½	1
	Vegetables	4	1	2
	Dressing, mayo or, margarine for the veggies	10	n.r	0
	Boiled or canned fruit	8	1	1
	Whipped cream	7	¼	½
16.00	Low protein bread	5	n.r	0
	Margarine, use generously	10	n.r	0
	Jam, marmalade, honey	10	n.r	0
	2 slices of bread	5	2	4
18.00	Margarine, use generously	10	n.r	0
	Sandwich spread	6	2	4
	A few veggie slices	4	<1	<1
	1 glass of rosehip cream	9	n.r	0
	Low protein bread	5	n.r	0
20.00	Margarine, use generously	10	n.r	0
	Jam, marmalade, honey or a few veggie slices	4 or 10	<1	<1
	½ apple	8	½	½

*Abbreviations*: *n.r* no restriction. *Comment:* each meal may include a beverage from food group 9 with no restriction

**Table 5:** Clinical presentation and follow up of a patient with LPD, Case 1

	Sept 1998	Feb 2000	May 2002	June 2004	May 2006	Mar 2008	Mar 2010	May 2013	Oct 2015
Weight (kg)	70,6*	72	72,5	72	75	73,5	73	73	71
GFR (mL/min)	30		28	23	20	16	15	13	10
FFMI (DXA or BIS]							Oct 2009 17,1		17,3
FMI (DXA or BIS)							6,8		5,2
S-Creatinine (μmol/L)	209	215	229	213	212	256	265	328	454
S-Urea (mmol/L)	12	9,4		12,2	11,2	15,6	13,9	16,9	23,3
s-Phosphate (mmol/L)	1,2			1,3	1,4	1,3	1,4	1,3	1,4
Protein intake (g/day: food diaries, 24 h recall or UNA)	0,7	0,8	0,8	0,75		0,73			0,7
Energy intake (kcal/day, food diaries)	1900	1900	2500	2600					2650

*Body Mass Index 21 kg/m^2^

*Abbreviations*: *GFR* glomerular filtration rate, *FFMI* fat free mass index, *FMI* fat mass index, *DXA* dual energy-x-ray absorptiometry, *BIS* bio-impedance spectroscopy, *UNA* urinary urea nitrogen appearance

Calculations: FMI = body fat (kg) / squared height in meters; FFMI = Body weight (kg) – body fat (kg)/squared height in meters

Reference values for FFMI and FMI according to the Swedish National Board of Health and Welfare’s cut-off values for malnutrition [23]: FFMI: Women <15 kg/m^2^, Men <17 kg/m^2^; FMI: Women <4 kg/m^2^, Men <2 kg/m^2^

The patient is now clearly entering a new phase in his CKD with a GFR at 10 ml /min. He has lost weight and has recently started adding one serving of low protein oral nutritional supplement (+ 6 g protein and 400 kcal) per day, which has helped him to regain 1 kg in weight and he is now (November 2015) back at 72 kg. He is still physically active, but not in the same range as earlier, he has a good appetite and denies all uremic symptoms at this point. Unfortunately his wife has been sick, which has made it harder for him to comply as perfectly as before with the diet during some periods for the past six months. For this reason he has monthly check-ups (telephone) of weight, appetite and uremic symptoms by the dietician in addition to the regular follow up by nephrologist at the out-ward clinic.

### Second patient case

A 68 years old man diagnosed with polycystic renal disease that since the early 70s attends regular visits to a nephrologist at the Renal Department at Karolinska University Hospital at Huddinge. Given his etiology, progression is slow. The patient is married and has three adult children. In February 2012 he was referred to a dietician for instruction of LPD treatment aiming at 0.6 g protein/Kg/day. The patient has experienced nausea and altered taste. His clinical presentation is shown in Table 6.

**Table 6:** Clinical presentation and follow up of a patient with LPD, Case 2

	1st visit February 2012	2nd visit April 2012	Last visit before starting dialysis June 2014
Weight (kg)	84,3	82,9	76
BMI (kg/m^2^)	29	28	25
eGFR (mL/min/1,73 m^2^)	19	16	12
P-Urea (mmol/L)	28,8	18,3	24,4
P-Creatinine (μmol/L)	389	401	777
P-Phosphate (mmol/L)	1,3	1,3	1,4
P-Potassium (mmol/L)	3,6	3,8	4,0
P-Albumin (g/L)	38	39	38
Pt(U)-Urea (mmol/d)		205 = 47 g protein	162 = 40 g protein

*Abbreviations*: *GFR* glomerular filtration rate, *BMI* body mass index

His laboratory values, especially the P-urea level indicates that he would likely benefit from a LPD. P-phosphate and P-potassium were within the reference ranges. No sign of inflammation (CRP > 5). Moreover the patient has a rather high BMI (29 kg/m^2^). A dietary recall was performed at his first visit and is shown below.

#### Dietary recall at 1^st^ encounter (February 2012)

***Breakfast***: 2 slices of toast, butter and marmalade, one cup of tea with honey, 2-3 dl of milk, sometimes 1½ dl yogurt

***Snack***: coffee

***Lunch***: milk and cereals, possibly one cheese sandwich or omelet (2 eggs) or residues or food at lunch restaurant ex one hamburger, 1 potato, gravy, vegetables, 1 rye bread with butter. Beverage: water with lemon.

***Snack***: coffee and cake or bun or cheese sandwich

***Dinner***: cooked meal: 2 dl pasta, 1.5-2 dl meat sauce or a piece of salmon (140 g), sauce, 1 potato, carrot, parsnip or tea with 2 cheese sandwiches. Beverage: beer or water with lemon

***Evening***: 1 fruit

*Estimated protein intake: 50-75 grams.*

Since the patient is slightly overweight, the protein restriction is adjusted according to his ideal body weight, which is 75 kg (weight according to BMI 25 + 25 % of weight difference): This means a daily protein intake around 45 g/day (0.6 x 75 = 45). BMI according to desirable weight was only used to calculate protein needs, not to restrict energy intake. The patient was not recommended to loose weight.

**Fig. 3:**
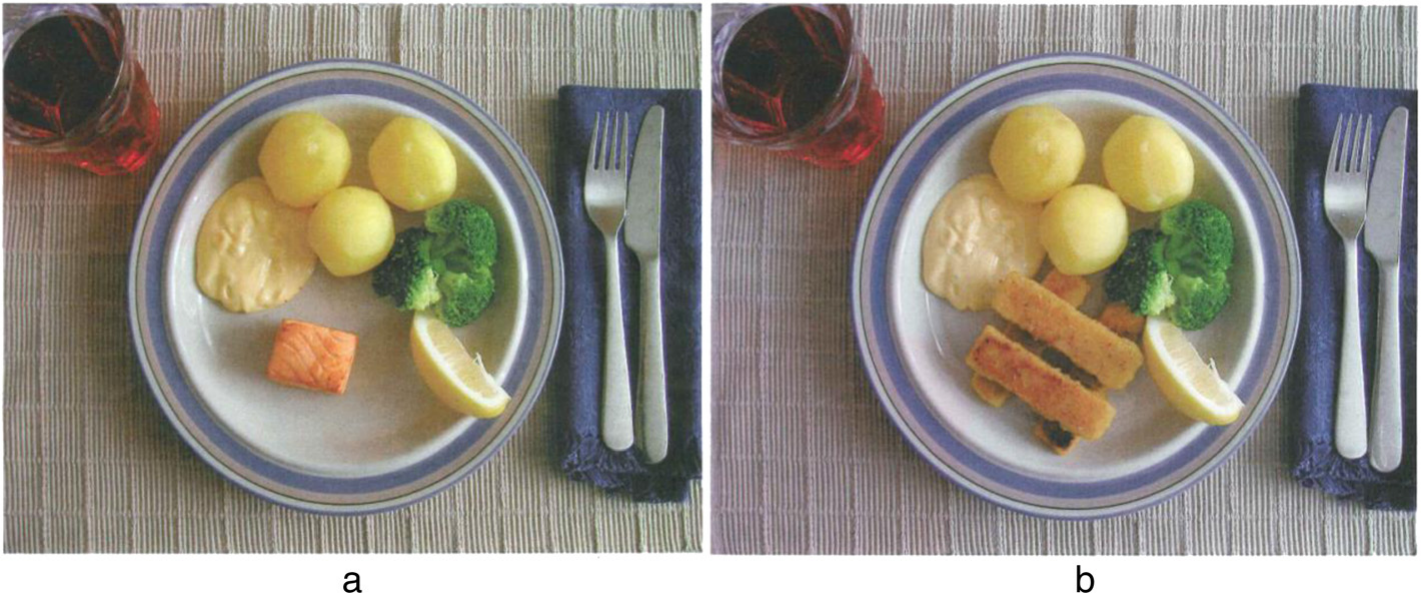
Graphical examples of meals providing approximately 10-12 g protein. The left plate (**a**): Salmon, sauce with crème fraiche and caviar. Right plate (**b**): Fish fingers with remoulade sauce

The dietician gives information about the purpose of the LPD, shows examples of low protein dishes such as diluted meat/fish dishes and cold cuts, as well as appropriate portion sizes of meat/fish. The patient is encouraged to plan his eating according to this regimen and for example distribute the 45 g of protein intake as follows: 10 g during breakfast, 12-15 g during lunch, 12-15 g during dinner and 5 g as snacks throughout the day. Graphical examples are presented to him regarding whole meals providing approximately 10-12 g protein (Fig. 3).

The dietician recommends him to replace some of the cheese with low protein spreads, like sausage/pate/cream cheese/jam, reduce the portion size of fish and restrict the consumption of milk/yogurt to a total of approximately 2-3 dl/day. The patient is encouraged to eat energy-dense foods to cover his estimated energy needs. The patient wants to try low protein pasta and other low protein products. Information about potassium and phosphorus (food content and potential harmful effects in CKD) was given. The levels of serum potassium and serum phosphate were satisfactory and the dietary recalls did not reveal any overconsumption of foods or beverages high in potassium or phosphorus. Protein rich foods like milk/yoghurt, cheese and meat are also rich in phosphorus so by eating less of them will reduce serum levels of both urea and phosphate. High levels of serum potassium is more common in hemodialysis, especially in anuric patients.

Some general advices for successful implementation of LPD are provided to the patient as take home messages:

- Add rice, potatoes, macaroni or spaghetti (ordinary or low protein pasta) to the food
- Add sauces to the food (use cream or crème fraiche instead of milk)
- Add cooked or raw vegetables. Put some margarine or butter on cooked vegetables and use oil-based dressing in the salad
- Milk is very rich in protein (1 liter = 35 g protein) and is therefore not recommended to drink with the meal. Recommended beverages are: beer, lemonade or water
- Add a dessert to the meal. For example canned fruit, fruit salad, rose hip soup, soup of berries with whipped cream
- Use standard 80 % fat margarine and dairy products instead of low fat products.
- Extend the snacks between meals (buns, biscuits, fruit)

One month later the patient returns. His laboratory values, presented in Table 6, indicated a progression of the CKD according to GFR, good adherence to the LPD according to Pt(U)-urea (urea excretion, mmol/d), stable p-phosphate and p-potassium and a weight reduction (1.4 kg in 2 months). A second dietary recall is requested, shown below:

#### Dietary recall at 2^nd^ visit (April 2012)

***Breakfast***: 2-3 slices of toast with butter and marmalade, tea, 1 dl of milk

***Snack***: coffee.

***Lunch***: 150 g pancakes, jam, cheese sandwich, Beverage: water with lemon

Snack: coffee and two biscuits with butter/cake.

***Dinner***: 3 fish fingers, rice, vegetables, remoulade sauce

Beverage: beer or water with lemon

***Evening***: Sometimes fruit

*Estimated protein intake: 40-45 grams*

The patient reported improved appetite. He found it easy to comply with the LPD at home but more difficult when visiting restaurants. The patient in this case was very motivated to follow the LPD but not so motivated to add extra fat and calories to his diet as can be seen in the above depicted dietary recall. Because of weight loss, he was recommended to use more fat (in sauces, as dressing to vegetables), to finish the dinner with a dessert and to eat more snacks in between meals. Further, an energy-dense emulsion (rich in unsaturated fatty acids) and enriched rosehip soup were prescribed. He was prescribed oral EAA and water-soluble vitamins. The patient visited the dietician another 2 times and had 3 telephone consultations. At his last visit before dialysis started, the patient reported early satiety and experienced food aversion especially to meat. He had lost more weight and his BMI was 25, still not alarming but weight loss together with uremic symptoms and declining GFR indicated that he was in need of dialysis. The patient was this time focused on how he was supposed to change dietary regimen when starting dialysis.
